# Mortality rates in Norwegian HEMS—a retrospective analysis from Central Norway

**DOI:** 10.1186/s13049-024-01202-4

**Published:** 2024-04-16

**Authors:** Stian Lande Wekre, Oddvar Uleberg, Lars Eide Næss, Helge Haugland

**Affiliations:** 1 Norwegian University of Science and Technology (NTNU), Trondheim, NO-7018 Norway; 2 Department of Emergency Medicine and Pre-Hospital Services, St. Olav’s University Hospital, Trondheim, Norway; 3 Department of Research and Development, Division of Emergencies and Critical Care, Oslo University Hospital, Oslo, Norway; 4 Department of Research and Development, Norwegian Air Ambulance Foundation, Oslo, Norway; 5 Department of Circulation and Medical Imaging, Faculty of Medicine and Health Sciences, Norwegian University of Science and Technology, Trondheim, Norway

**Keywords:** Anesthesiology, Cohort studies, Emergency medical services, Mortality, Outcome assessment, Health care, Retrospective studies, Severity of illness index, Trauma severity indices

## Abstract

**Background:**

Helicopter Emergency Medical Services (HEMS) provide rapid and specialized care to critically ill or injured patients. Norwegian HEMS in Central Norway serves an important role in pre-hospital emergency medical care. To grade the severity of patients, HEMS uses the National Advisory Committee for Aeronautics’ (NACA) severity score. The objective of this study was to describe the short- and long term mortality overall and in each NACA-group for patients transported by HEMS Trondheim using linkage of HEMS and hospital data.

**Methods:**

The study used a retrospective cohort design, aligning with the STROBE recommendations. Patient data from Trondheim HEMS between 01.01.2017 and 31.12.2019 was linked to mortality data from a hospital database and analyzed. Kaplan Meier plots and cumulative mortality rates were calculated for each NACA group at day one, day 30, and one year and three years after the incident.

**Results:**

Trondheim HEMS responded to 2224 alarms in the included time period, with 1431 patients meeting inclusion criteria for the study. Overall mortality rates at respective time points were 10.1% at day one, 13.4% at 30 days, 18.5% at one year, and 22.3% at three years. The one-year cumulative mortality rates for each NACA group were as follows: 0% for NACA 1 and 2, 2.9% for NACA 3, 10.1% for NACA 4, 24.7% for NACA 5 and 49.5% for NACA 6. Statistical analysis with a global log-rank test indicated a significant difference in survival outcomes among the groups (*p* < 2⋅10^− 16^).

**Conclusion:**

Among patients transported by Trondheim HEMS, we observed an incremental rise in mortality rates with increasing NACA scores. The study further suggests that a one-year follow-up may be sufficient for future investigations into HEMS outcomes.

**Supplementary Information:**

The online version contains supplementary material available at 10.1186/s13049-024-01202-4.

## Background

Patients transported by Helicopter Emergency Medical Services (HEMS) have a high short-term (30-day) mortality as HEMS is often dispatched to critically ill or injured patients [1]. Recent studies have also found increased mortality after one and three years. In a population-based study conducted in Denmark, a one-year mortality of 19.5% was observed among all airlifted patients [2], while a comparable study from Finland revealed a three-year mortality of 36.5% among patients treated by the HEMS [3]. How and to what extent HEMS impact mortality compared to Ground Emergency Medical Services (GEMS) is however still under discussion [4–8]. While HEMS has proven effective in improving outcomes and lowering mortality for trauma and traumatic brain injury patients [6, 9, 10], its impact on less critically ill patients remains a debated topic [11, 12]. These studies do however come from an American system, were the staffing of HEMS and GEMS is quite similar, primarily consisting of emergency medical technicians and paramedics. Thus, these studies mainly focus on reduced transport times.

HEMS in Norway, as well as much of Europe, differ from GEMS as they are mostly staffed by an anesthesiologist. The indication for HEMS use is therefore not only determined by the need for fast transportation, but also if the mission calls for specialized medical surveillance or treatment [13]. The role and benefits of physician-staffed emergency medical services (P-EMS) are also actively discussed. While bringing advanced medical therapy to the scene may enhance survival [14], concerns linger about potential transport delays, a factor linked to increased mortality in some studies [15]. Since operating HEMS comes with substantial costs, it is crucial to ensure that the service is dispatched to where it can be most effective. This not only improves patient care but also maximizes the cost-effectiveness of this specialized service. For this reason, continuous data linkage between HEMS data and hospital data records may enhance the quality of care throughout the chain of care through quality initiatives. Systematically assessment of long-term mortality is rare, as linkage between data records is mainly performed on a project-by-project basis [16].

To identify and categorize the severity of a patient’s condition, the Norwegian HEMS utilizes National Advisory Committee for Aeronautics (NACA)– score [17, 18]. The patients are assigned a NACA-score after each mission by the treating physician. The score is dependent on the clinical and subjective evaluation from this physician. The scale ranges from 0 to 7, where 0 represents no injury or disease and 7 describes a patient that died during the mission. The description for each score is showed in Table 1. The NACA score is not intended nor used as a triage system to control dispatch of HEMS. Some also argue that it is not suited for epidemiological studies or quality control because of its highly subjective nature [19, 20]. However, Raatiniemi et al. showed that the NACA score is good at predicting mortality [1]. They argue that it is still useful for comparing how severe cases are, given that it reasonably predicts patient outcomes and shows low variability among physicians.

Reduction in mortality is one of the most common variables that describe quality of care [21]. The objective of this study was to describe the short- and long term mortality overall and in each NACA-group for patients transported by HEMS Trondheim using linkage of HEMS and hospital data.

**Table 1:** The national advisory committee on aeronautics (NACA)—score, used in Norwegian HEMS to classify severity of injury or sickness [22]

NACA 0	No injury or disease
NACA 1	Injuries/diseases without any need for acute physician’s care
NACA 2	Injuries/diseases requiring examination and therapy by a physician, but hospital admission is not indicated
NACA 3	Injuries/diseases without acute threat to life but requiring hospital admission
NACA 4	Injuries/diseases that can possibly lead to deterioration of vital signs
NACA 5	Injuries/diseases with acute threat to life
NACA 6	Injuries/diseases transported after successful resuscitation of vital signs
NACA 7	Lethal injuries or diseases (with or without resuscitation attempts)

## Methods

The study is a retrospective cohort study. The study follows the ‘Strengthening the reporting of observational studies in epidemiology’ (STROBE) recommendations for reporting of observational cohort studies [23].

### Study setting

Norway has the second lowest population density in Europe, where only Iceland is less densely populated [24]. The long distances in combination with harsh climate and difficult terrain [24] make Norwegian HEMS important in the care for critically ill patients [25]. It allows for shorter patient transportation times, the transport of advanced medical equipment and personnel to the point of injury and access to sites that would otherwise be inaccessible [7, 14, 26]. The Norwegian National Air Ambulance Services is publicly financed and consist of both helicopters and fixed wing aircrafts. The helicopter service is operated by the Norwegian Air Ambulance Helicopter and they operate 14 helicopters at 13 bases. All helicopters are staffed by a pilot, a HEMS crew member and a consultant anesthesiologist. Moreover, the Norwegian Air Force and Canadian Holding Company (CHC), a private contractor, operates search and rescue helicopters at a total of seven bases on mainland Norway. These helicopters may be used to fly air ambulance missions when available [27].

The Central Norway Regional Health authority serves approximately 700 000 inhabitants over an area of 56 000 square kilometers [28]. The region’s HEMS consists of two bases, one in Trondheim and one in Ålesund. In addition, a search and rescue helicopter is stationed at Ørland Air Force Base. To simplify the data-collection process and avoid potential bias from difference in the stations staffing we only included data from the Trondheim HEMS base.

### Data collection

We used data from the emergency medicine communications central’s (EMCC) database AMIS (Akuttmedisinsk informasjonssystem, CSAM health AS, Oslo, Norway), the HEMS’ database LABAS (Normann IT, Trondheim, Norway) and the hospital Patient Administrative System (PAS). As part of a continuous quality scheme at our base, these data sources are updated once every 24 h. AMIS is a documentation system that is used by every EMCC in Norway. It contains data on the time of the alarm, what resources were dispatched, response times, and patient data. LABAS is the dedicated operational database and medical record generator used by Norwegian HEMS.

**Fig. 1:**
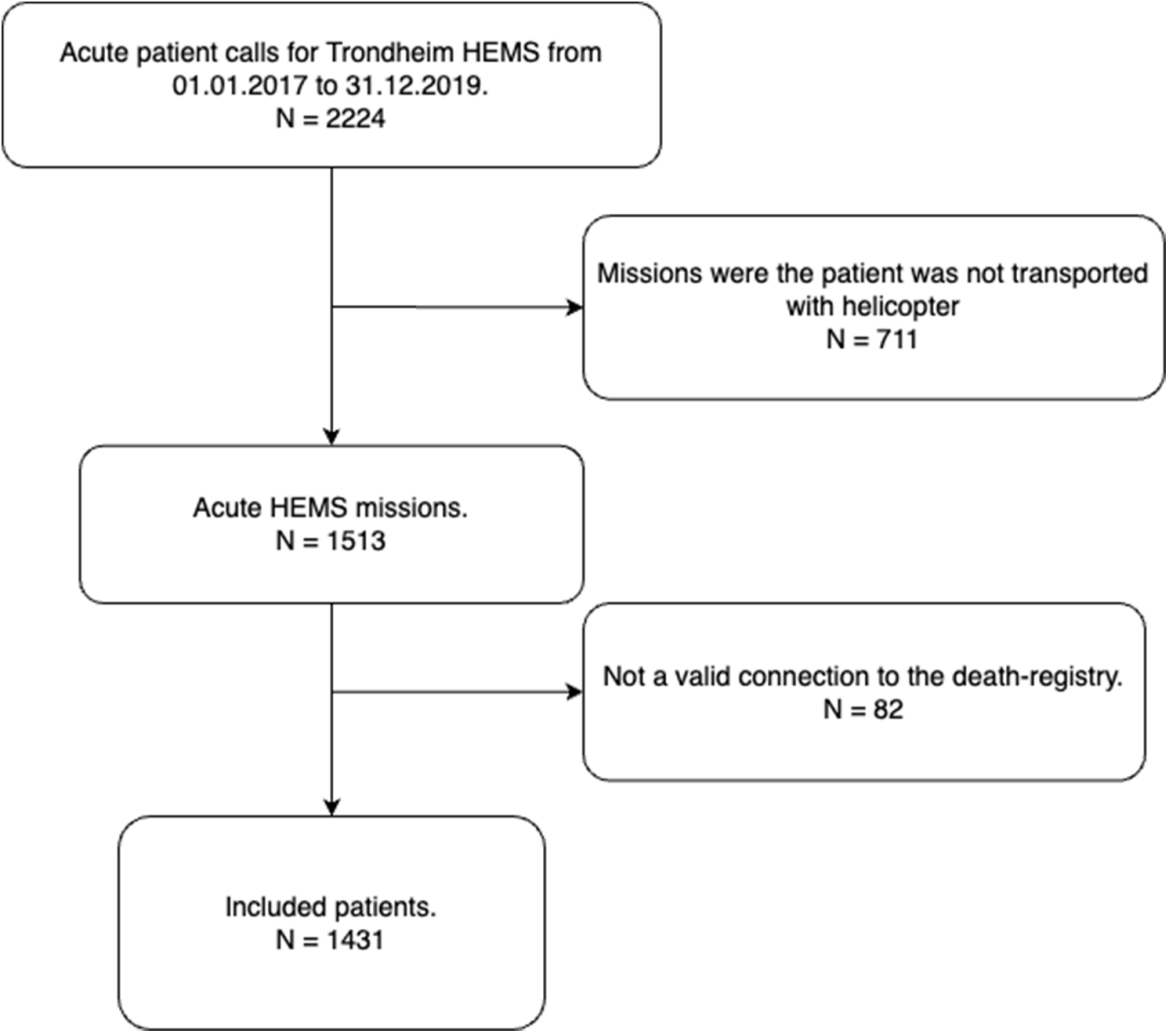
Flow chart showing the population selection

From LABAS we extracted information on the date and time of the alarm, patients` gender, date of birth, age, vehicle type used (e.g. helicopter or rapid response car), municipality, NACA-score, and AMIS-number. The AMIS-number is an autogenerated sequence number assigned by the EMCC and is unique for each mission. By using the AMIS-number, the data was linked through the AMIS database to obtain a personal identification number used to extract time of death from PAS and the Norwegian Cause of Death Registry. Data from all completed primary missions (i.e. responding to acute illness or injury out of hospital) from the HEMS base in Trondheim, Norway, between 01.01.2017 and 31.12.2019 were extracted. Exclusion criteria included missions where no helicopter was deployed (cancelled missions and missions responded to by car), missions with no air-lifted patients (patient transported by GEMS, or by other means), non-acute missions, secondary missions (transporting patients between hospitals) and missions without a valid connection to the PAS (due to no patient contact or documentation error). Thus, only data from patients that were airlifted were included, as the scope of this study was to analyze the mortality after receiveing treatment in a HEMS-setting. Flow chart for the data collection is shown in Fig. 1.

### Statistical analysis

Descriptive statistics is presented as median with interquartile range for continuous data that are not normally distributed. Mortality and survival rates are analyzed in each NACA-group using the survival (version 3.5-7), survminer (version 0.4.9) and ggsurvfit (version 0.3.1) packages for R [29–31]. The mortality is shown graphically using a Kaplan Meier plot with 95% confidence intervals and calculated as cumulative mortality rate at day 1, day 30, one year (day 365) and three years (day 1095). To analyze for statistically significant difference between the groups, a log-rank test is used. In the case that the generalized log-rank test is statistically significant, a pairwise comparison of the individual groups is done, also using the log-rank test. Statistical analysis was performed using R (version 4.3.1 (2023-06-16)) in RStudio [32].

## Results

### General results

In the study period, Trondheim HEMS responded to 2 224 calls. Of these, 827 were excluded because the patients were responded to by car (*n* = 711) or due to a lack of connection to the death registry (*n* = 82) (Fig. 1). A total of 1431 patients were eligible for inclusion.

The characteristics for the included patients can be found in Table 2.

**Table 2:** Summary statistics

	Total, n	Males, n (%)	Females, n (%)	Median age in years (IQR)
NACA 1	11	7 (64%)	4 (36%)	59 (19/74)
NACA 2	69	33 (48%)	36 (52%)	31 (11/54)
NACA 3	450	299 (66%)	151 (34%)	41 (17/61)
NACA 4	506	334 (66%)	172 (34%)	63 (48/73)
NACA 5	190	124 (65%)	66 (35%)	64,5 (55/75)
NACA 6	107	73 (68%)	34 (32%)	64 (56/73)
NACA 7	98	73 (75%)	25 (25%)	66,5(53/75)
Overall	1431	943 (66%)	488 (34%)	58 (33/71)
**By age group**				
0–17	207	120 (58%)	87 (42%)	
18–64	675	447 (66%)	228 (34%)	
65–79	415	299 (72%)	116 (28%)	
≥ 80	134	77 (58%)	57 (42%)	
**Prehospital procedures**				
Intubation	185	128 (69%)	57 (31%)	
Vasoactive drugs	218	148 (68%)	70 (32%)	

### Mortality

**Fig. 2:**
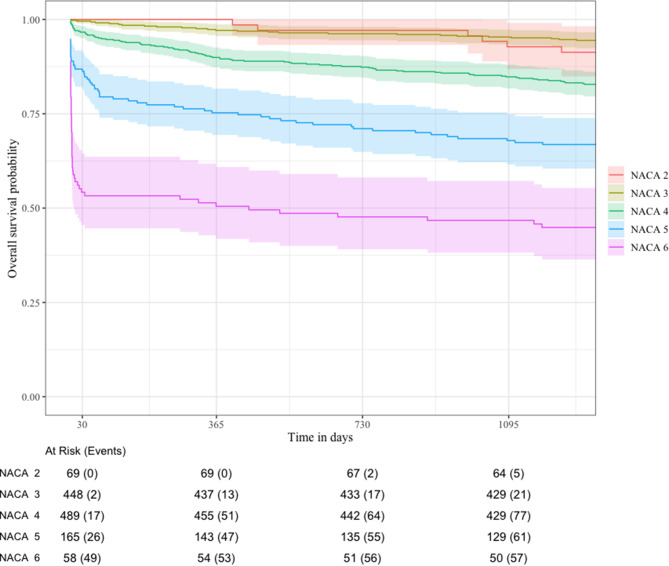
Survival plot by NACA-score (with 95% CI) NACA group 1 and 7 are excluded to simplify the plot. NACA 1 had zero deaths within the follow-up period, and NACA 7 are by definition deceased

Overall mortality, across all NACA groups, at day one, 30, one year and three years were 9.9%, 13.2%, 18.2% and 22.2%, respectively. The cumulative mortality rates in each NACA-group are shown (Table 3). Mortality increased with NACA score at all time points, except for NACA score 1 and 2 at day one, 30 and one year, as none of these patients died within the first year. A graphical representation of the mortality rates is shown in the Kaplan-Maier plot (Fig. 2). The NACA 1 group had no deaths in the entire follow-up period and patients in the NACA 7 group were by definition deceased on the day of the incident (day 0). We observed an increase in mortality for every increase in NACA score at all time points, except for NACA group 2 and 3 at the three-year time point (Fig. 2).

There was a statistically significant difference among the groups, with a global log-rank test value of *p* < 2⋅10^− 16^. We therefore proceeded with pairwise comparison of the groups. Test results showed that NACA-group 1 and 2 only were significantly different from the groups with a NACA-score of 5 or higher and 4 or higher, respectively. All other comparisons showed a significant difference. The p-values for all tests can be found in Table 4.

**Table 3:** Cumulative mortality rates (95% CI)

	1 day	30 days	1 year (365 days)	3 years (1095 days)
NACA 1	0% (0/0)	0% (0/0)	0% (0/0)	0% (0/0)
NACA 2	0% (0/0)	0% (0/0)	0% (0/0)	7,2% (0,9/13,2)
NACA 3	0% (0/0)	0.4% (0/1.1)	2.9% (1.3/4.4)	4.7% (2.7/6.6)
NACA 4	1% (0.1/1.9)	3.4% (1,8/4,9)	10.1% (7.4/12.7)	15.2% (12/18.3)
NACA 5	10% (5.6/14.1)	13.7% (8.7/18.4)	24.7% (18.3/30.6)	32.1% (25.1/38.4)
NACA 6	20.6% (12.5/27.9)	45.8% (35.5/54.5)	49.5% (39.1/58.2)	53.3% (42.8/61.8)
NACA 7	100% (100/100)	100% (100/100)	100% (100/100)	100% (100/100)
Overall	10.1% (8.5/11.6)	13.4% (11.6/15.5)	18.5% (16.3/20.3)	22.3% (20.1/24.4)

**Table 4:** P-values for pairwise comparison of NACA-groups with Log-Rank test

	NACA 1	NACA 2	NACA 3	NACA 4	NACA 5	NACA 6
NACA 2	0.3526					
NACA 3	0.3884	0.6847				
NACA 4	0.1213	0.0174*	3.8e-10*			
NACA 5	0.0262*	5.5e-06*	< 2e-16*	4.6e-07*		
NACA 6	0.0019*	1.8e-11*	< 2e-16*	< 2e-16*	4.0e-05*	
NACA 7	< 2e-16*	< 2e-16*	< 2e-16*	< 2e-16*	< 2e-16*	< 2e-16*

*p < 0.05

## Discussion

In our study we observed that by using data linkage of HEMS and hospital data, we were able to describe mortality among patients transported by HEMS. We found that short- and long term mortality increases with higher NACA-score. This supports previous studies that have found NACA score to be a predictor of mortality [1, 18, 22].

From the pairwise comparison with the log-rank test we also know that the difference between most of the groups is statistically significant. However, we found no difference in mortality between the groups with a NACA score of 1, 2 or 3. This is most likely due to the very small sample size of patients with NACA 1 and 2, with 11 and 69 respectively, and very few deaths in the follow-up period. We also believe that this causes the descrepency at the three-year time point were the NACA 2 group has a higher mortality than the NACA 3 group. As there are so few in the NACA 2 group, the 5 deaths that occur between year one and three makes a large impact on the mortality rate and must be interpereted cautiously.

Our study aligns with earlier research on mortality in some NACA groups but shows differences in others. Stratifying on NACA groups, Bonatti et al. found a 30-day mortality of 2.6%, 14.4% and 87.2% for NACA score 4, 5 and 6 respectively, and 0% for the other groups [22]. This closely resembles our findings for the NACA 4 and the NACA 5 group but is near the double of the mortality in the NACA 6 group in our study. As the NACA score is a subjective evaluation from the treating physician, the difference for the NACA 6 group could be due to the differences in interpretation of the scale [33]. There may also be differences in what requests the services respond to, and in what cases the physician decides that the use of HEMS is warranted. In Norwegian HEMS, responding to a request from the EMCC is at the attending physician’s discretion. Norwegian HEMS has been found to have a higher proportion of aborted missions than similar services, due to the attending physician deciding the necessity of a HEMS dispatch [34]. Another reason for the lower mortality rate in this study may be due to the general improvements seen in medical technology and medical care. There is more than 20 years between the studies, thus increasing the chances of survival for similar medical conditions. One must also consider the demographical differences, with Norwegian HEMS generally serving a rural population with long flight times, and possibly different dispatch criteria [35].

In a study from Wills et al. they found a 30-day mortality of 0.7%, 12.2% and 50% for NACA score 4, 5 and 6 respectively, for 427 trauma patients admitted to the emergency room [18]. Somewhat lower than what we found for NACA group 4 and 5, but higher for NACA group 6. However, this study included only trauma patients and the study population is relatively small. Trauma patients have been found to have lower mortality than nontrauma patients in the Danish HEMS [36]. The results may therefore be skewed towards a lower mortality. Wills et al. also analyzed one year mortality and found this to be 0.6%, 2.8%, 21.9% and 50% for NACA group 3, 4, 5 and 6 respectively. This very much resembles our findings but have only a third of the mortality of the NACA 3 and NACA 4 group in our study. This again may be due to local variation in interpretation of the scale or different study populations.

The cumulative mortality for the patients in our study is substantial and keeps increasing long after the initial incident. This is in line with two other studies looking at an unselected population being transported by HEMS in Finland and Denmark [2, 3]. Alstrup et al. found a cumulative mortality rate of 8.2%, 16.2% and 19.5% among airlifted patients on day 1, 30 and 365 respectively [2]. This is comparable to our results. Björkman et al. only report the cumulative mortality rates at 3 years, found to be 36.5% [3]. This is considerably higher than what we found in our study population. Finnish HEMS closely resembles that of Norway in several ways [35] but have also previously been found to have higher mortality rates than the Norwegian service [37].

Even though mortality increases for a long period after the incident, the increase from one until three years is small. This suggests that the effect of the injury or sickness treated by HEMS may no longer have a significant impact on the patients’ health, and that other causes of death may be just as likely. We therefore believe that a one-year follow-up period is enough for future studies on this subject.

The strength of our study is the integrated data linkage between the HEMS documentation system, and other hospital medical records. The PAS extracts time of death from the Norwegian Cause of Death Registry daily, allowing us to include a large population with quality assured mortality data with a 3-year follow up. To the best of our knowledge, this is the first time a study describes the 3-year mortality in different NACA groups.

The limitations of our study are lack of information about cause of death, comorbidities, and certain hospital data. This decreases the validity of the long-term data, as we are unable to ensure a correlation between their contact with HEMS and cause of death. Moreover, only including data from one HEMS base limits the possibility to generalize our findings, thus limiting the external validity of the study. 82 patients were excluded from the study due to a non-valid connection to the Norwegian Cause of Death Registry. We found that among these are persons who were admitted to a hospital outside of the Central Norway Regional Health Authority and people without a Norwegian personal identification number (PIN). This would mostly be tourists and small children who have not yet received a PIN.

Future studies may want to include data on cause of death, in-hospital diagnosis, and other markers on quality of life, for instance welfare benefits, to investigate other long-term outcomes in each NACA group.

## Conclusions

Among patients transported by Trondheim HEMS, we observed an incremental rise in mortality rates with increasing NACA scores. One year after the incident, the cumulative mortality rate in NACA group 3–6, nearly doubles with every increase in NACA score. The study further suggests that a one-year follow-up may be sufficient for future investigations into HEMS outcomes.

### Electronic supplementary material

Below is the link to the electronic supplementary material.

Supplementary Material 1

## Data Availability

The data for this study is not publicly available, but available from Central Norway Regional Health Authority upon reasonable request. R-code used in the data analysis is available from the corresponding author upon reasonable request.

## Abbreviations

CHC: Canadian Holding Company
EMCC: Emergency Medical Communication Central
GEMS: Ground Emergency Medical Service
HEMS: Helicopter Emergency Medical Service
NACA: National Advisory Committee for Aeronautics
PAS: Patient Adminstrative System

## References

[1] Raatiniemi L, Mikkelsen K, Fredriksen K, Wisborg T (2013). Do pre-hospital anaesthesiologists reliably predict mortality using the NACA severity score? A retrospective cohort study. Acta Anaesthesiol Scand.

[2] Alstrup K, Petersen JAK, Sollid S, Johnsen SP, Rognås L (2020). Mortality and hospitalisation in the Danish Helicopter Emergency Medical Service (HEMS) population from 2014 to 2018: a national population-based study of HEMS triage. BMJ Open.

[3] Björkman J, Laukkanen-Nevala P, Olkinuora A, Pulkkinen I, Nurmi J (2021). Short-term and long-term survival in critical patients treated by helicopter emergency medical services in Finland: a registry study of 36 715 patients. BMJ Open.

[4] Galvagno SM Jr, Sikorski R, Hirshon JM, Floccare D, Stephens C, Beecher D et al. Helicopter emergency medical services for adults with major trauma. Cochrane Database Syst Reviews. 2015(12). 10.1002/14651858.CD009228.pub3 PMC8627175 26671262

[5] Tsuchiya A, Tsutsumi Y, Yasunaga H (2016). Outcomes after helicopter versus ground emergency medical services for major trauma–propensity score and instrumental variable analyses: a retrospective nationwide cohort study. Scand J Trauma Resusc Emerg Med.

[6] Andruszkow H, Lefering R, Frink M, Mommsen P, Zeckey C, Rahe K (2013). Survival benefit of helicopter emergency medical services compared to ground emergency medical services in traumatized patients. Crit Care.

[7] Mommsen P, Bradt N, Zeckey C, Andruszkow H, Petri M, Frink M (2012). Comparison of helicopter and ground emergency medical service: a retrospective analysis of a German rescue helicopter base. Technol Health Care.

[8] Alstrup K, Rognås L, Sollid S, Johnsen SP, Valentin JB, Petersen JAK (2021). Association of Helicopter vs Ground Emergency Medical Transportation with 1-Year mortality in Denmark. JAMA Netw Open.

[9] Taylor C, Jan S, Curtis K, Tzannes A, Li Q, Palmer C (2012). The cost-effectiveness of physician staffed Helicopter Emergency Medical Service (HEMS) transport to a major trauma centre in NSW, Australia. Injury.

[10] Galvagno SM, Haut ER, Zafar SN, Millin MG, Efron DT, Koenig GJ (2012). Association between helicopter vs ground emergency medical services and survival for adults with major trauma. JAMA.

[11] Bulger EM, Guffey D, Guyette FX, MacDonald RD, Brasel K, Kerby JD (2012). Impact of prehospital mode of transport after severe injury: a multicenter evaluation from the Resuscitation outcomes Consortium. J Trauma Acute Care Surg.

[12] Davis DP, Peay J, Serrano JA, Buono C, Vilke GM, Sise MJ (2005). The impact of aeromedical response to patients with moderate to severe traumatic brain injury. Ann Emerg Med.

[13] Luftambulansetjenesten HF. Retningslinjer for bruk av luftambulanse. 2009.

[14] Hesselfeldt R, Steinmetz J, Jans H, Jacobsson ML, Andersen DL, Buggeskov K (2013). Impact of a physician-staffed helicopter on a regional trauma system: a prospective, controlled, observational study. Acta Anaesthesiol Scand.

[15] Yamamoto R, Suzuki M, Yoshizawa J, Nishida Y, Junichi S (2021). Physician-staffed ambulance and increased in-hospital mortality of hypotensive trauma patients following prolonged prehospital stay: a nationwide study. J Trauma Acute Care Surg.

[16] Andrew E, Cox S, Smith K (2022). Linking Ambulance records with Hospital and Death Index Data to evaluate patient outcomes. Int J Gen Med.

[17] Tryba M, Brüggemann H, Echtermeyer V (1980). Klassifizierung Von erkrankungen und verletzungen im notarztrettungssystemen. Notfallmedizin.

[18] Weiss M, Bernoulli L, Zollinger A (2001). [The NACA scale. Construct and predictive validity of the NACA scale for prehospital severity rating in trauma patients]. Anaesthesist.

[19] Schlechtriemen T, Burghofer K, Lackner CK, Altemeyer KH (2005). Validierung Des NACA-Score anhand objektivierbarer parameter. Notf Rettungsmedizin.

[20] Lackner CK, Altemeyer KH (2005). Quo vadis NACA-Score?. Notf Rettungsmedizin.

[21] Goodacre S, Campbell M, Carter A (2015). What do hospital mortality rates tell us about quality of care?. Emerg Med J.

[22] Bonatti J, Göschl O, Larcher P, Wödlinger R, Flora G (1995). Predictors of short-term survival after helicopter rescue. Resuscitation.

[23] von Elm E, Altman DG, Egger M, Pocock SJ, Gøtzsche PC, Vandenbroucke JP (2014). The strengthening the reporting of Observational studies in Epidemiology (STROBE) Statement: guidelines for reporting observational studies. Int J Surg.

[24] Meld. St. 20 (2020–2021). National Transport Plan 2022–2033. In: Transport NMo, editor. 2021.

[25] Helsedirektoratet. The Nordic emergency Medical Services Benchmarking Report. 2018.

[26] Svenson JE, O’Connor JE, Lindsay MB (2006). Is air transport faster? A comparison of air versus ground transport times for interfacility transfers in a regional referral system. Air Med J.

[27] Luftambulansetjenesten. About the National Air Ambulance Services of Norway 2022 [updated 19. dec, 2022. https://www.luftambulanse.no/om-oss/about-the-national-air-ambulance-services-of-norway/.

[28] NOU. 2016: 25. Organisering og styring av spesialisthelsetjenesten - Hvordan bør statens eierskap innrettes framover? In: Ministry of Health and Care Services, editor. 2016.

[29] Therneau TM (2023). Survival: a Package for Survival Analysis in R. 3.5-7 ed.

[30] A K, M K. P B. survminer: Drawing Survival Curves using ‘ggplot2’. 0.4.9 ed2021.

[31] C DSMB, T T. F, S H,. ggsurvfit: Flexible Time-to-Event Fig. 0.3.1 ed2023.

[32] R Core Team (2023). R: a Language and Environment for Statistical Computing.

[33] Schlechtriemen T, Burghofer K, Lackner CK, Altemeyer K (2005). Validation of the NACA score based on objectifiable parameters: analysis of 104,962 primary air rescue missions in 1999–2003: Untersuchung an 104.962 Primäreinsätzen Der Jahre 1999–2003 Aus Der Luftrettung. Notf Rettungsmedizin.

[34] Østerås Ø, Brattebø G, Heltne JK (2016). Helicopter-based emergency medical services for a sparsely populated region: a study of 42,500 dispatches. Acta Anaesthesiol Scand.

[35] Krüger AJ, Skogvoll E, Castrén M, Kurola J, Lossius HM (2010). Scandinavian pre-hospital physician-manned Emergency Medical services–same concept across borders?. Resuscitation.

[36] Sørensen LM, Rognås L, Alstrup K (2021). Trauma Versus Nontrauma patients treated by the Danish Helicopter Emergency Medical Service: a Register-based study of patient characteristics, hospitalization, and Mortality. Air Med J.

[37] Haugland H, Olkinuora A, Rognås L, Ohlén D, Krüger A (2020). Mortality and quality of care in nordic physician-staffed emergency medical services. Scand J Trauma Resusc Emerg Med.

