# Supplementary material for: Mortality rates in Norwegian HEMS—a retrospective analysis from Central Norway
Source: Scand J Trauma Resusc Emerg Med. 2024 Apr 16;32:29. doi: 10.1186/s13049-024-01202-4 (PMC11022357; doi:10.1186/s13049-024-01202-4)
Supplement: Supplementary file 1 — Supplementary Material 1 [file 13049_2024_1202_MOESM1_ESM.pdf]

Response to Reviewers

Dear Editor Dr Rognås,
Dear Sirs and Madams

Thank you for giving us the opportunity to submit a revised version of the manuscript "Mortality rates in Norwegian HEMS – a retrospective analysis from Central Norway" for publication in the Scandinavian Journal of Trauma, Resuscitation and Emergency Medicine. We would like to express our gratitude for the time and effort that You and the reviewers have put into your feedback and comments on our manuscript. You have provided valuable improvement to the text, which we believe have improved the presentation of our results. Our response to the comments is provided below with a point-by-point response. All page numbers refer to the revised manuscript with tracked changes.

Editors`comments:

- Page 2 / line 49:

Authors response: No comparisons to other systems were performed. We changed the wording to better reflect the aim of the study (Page 2 / line 51-52).

- Page 2 / lines 54 – 56:

Authors response: The text was corrected and shortened both in the abstract and the conclusion, so that it better describes our findings (Page 2 / lines 51-54). We agree that the use of NACA does not affect the prioritizing of patients per se as NACA scoring is provided after mission completion.

- Page 3 / line 68:

Authors response: Thank you for a great reference suggestion. We agree that this adds important knowledge to the field and have added it to the to the reference list (new reference #8) (Page 3 / line 68).

- Page 3 / line 86:

Authors response: Thank you for the correction, the word "-score" has been added to the text (Page 3 / line 89).

- Page 4 / lines 96 – 101:

Authors response: This has been shortened and corrected so that the end of the introduction matches the abstract (Page 4 / lines 99-101).

- Page 5 / line 119:

Authors response: The correct indefinite article "a" was added (Page 5 / line 119).

- Page 5 / line 120:

<u>Authors response:</u> We added a more comprehensive explanation of the Norwegian rescue helicopters (Page 5 /lines 120-122).

- Page 5 / line 132 – 133:

<u>Authors response:</u> The sentence was rephrased in a more proper English language style (Page 5 / lines 135-136).

- Page 5 / line 133:

<u>Authors response:</u> Thank you for your correction and «medical record» was indeed what we intended to write (page 5 / lines 135-136).

- Page 5 / line 136:

<u>Authors response:</u> We added the missing word «used» (Page 5 / line 138).

- Page 5 / line 143:

<u>Authors response:</u> "Was" was corrected to "were" (Page 5 / line 144).

- Page 5 / lines 144 – 145:

<u>Authors response:</u> The scope of the study was to examine the short- and long-term mortality for patients whom were transported by HEMS. Retrospectively we acknowledge that it would have been of great interest to also include the patients who were responded to by car, and that this should be done in future research. We have elaborated on this in the revised manuscript (Pages 5-6 / lines 146-151).

- *<u>Page 6 / line 164:</u>*

<u>Authors response:</u> Thank you for pointing out this and we agree that the wording could lead to misinterpretation. We have changed the wording to avoid this misunderstanding (Page 6 / line 167).

- Page 6 / line 167:

<u>Authors response:</u> Table 2 has been updated with the percentages for each group (Pages 6-7).

- Page 8 / line 191:

<u>Authors response:</u> We have moderated our claims to better reflect our findings in the study (Page 8 / lines 194-196).

- <u>Page 8 / lines 192 – 197:</u>

<u>Authors response:</u> The summary of the results was removed to avoid unnecessary repetition (Page 8 / lines 194-197).

- Page 8 / line 211:

<u>Authors response:</u> We added the missing wording "mortality in the" to the manuscript (Page 8 / line 210).

- Page 9 / line 214:

<u>Authors response:</u> We thank you for your suggestions on better lingual phrasing and have hopefully reworded the sentence to make it less «Norwenglish" (Page 9 / lines 213-214).

- Page 9 / line 215 first:

<u>Authors response:</u> We added the missing comma (Page 9 / line 214).

- Page 9 / line 215 last:

<u>Authors response:</u> Thank you for this timely comment. It could be that such a system skews the distribution of NACA-scores towards higher values in the population attended by HEMS. Which we would argue, is positive, in terms of using a costly HEMS system to those patients who mostly benefit a fast and advanced medical treatment capacity, with increased cost-effectiveness. Though, the discussion of cost-effectiveness of HEMS is vastly beyond the scope of this article. Regarding our findings, we did not investigate whether this practice would have an actual effect on the mortality rates. However, we have no indication, that this would affect mortality, though we agree that the proportions of NACA score with higher values might be more prevalent in our study compared to similar studies.

- Page 9 / line 217:

<u>Authors response:</u> Indeed. We therefore believe that this was worth elaborating on in our manuscript. Please see the comments above and our revised manuscript.

- Page 9 / line 227:

<u>Authors response:</u> This is a very good point. We added a reference (new reference #36), explaining how the differences between the studies may be explained (Page 9 / line 227-228).

- Page 9 / lines 236 – 237:

<u>Authors response:</u> We changed the wording according to your suggestion (Page 9 / line 237).

- Page 9 / lines 242 – 243:

<u>Authors response:</u> We did not find a suitable reference and the statement has therefore been removed.

- Page 10 / lines 261 – 262:

<u>Authors response:</u> Thank you for this comment. After discussing this subject further among the authors, we found that this statement cannot be substantiated by further references and we cannot conclude that the excluded cases share a similar mortality. We have for this reason removed this claim.

- Page 10 / line 268:

<u>Authors response:</u> We changed the wording to avoid using a comparing word without a comparison (page 10 / lines 265-269). In addition, the conclusion was also partially rephrased to better comply with the comments presented by reviewer 2.

- Page 10 / lines 272 – 273:

<u>Authors response:</u> We moderated our conclusion in the revised manuscript to better reflect our findings in the study (page 10 / lines 265-269).

Reviewer #1 comments:

- I agree with the statement by the authors, this article provides valuable reference values for services using the NACA score.

<u>Authors response:</u> We thank you for this kind response.

- In the "abbreviations" part, NACA is not correct. National Advisory Committee for Aeronautics is the correct name.

<u>Authors response:</u> Thank you for bringing this to our attention. We have corrected the abbreviation to reflect the accurate name, 'National Advisory Committee for Aeronautics (NACA)', in the revised manuscript.

- Thank you for your study, we plan to use the results once published.

<u>Authors response:</u> We look forward to seeing how our study may be utilized in your future research.

Reviewer #2:

Thank you for the opportunity to review this retrospective study about mortality for HEMS patients transported by Trondheim HEMS and its association with NACA score.
The study reports results from a single region from which a survival analysis has been made overall and according to NACA score. Even though I acknowledge the great work of the authors, I find the study a little "thin" and mainly interesting for local stakeholders.

- The knowledge gap that needs to be solved in this paper does not emerge clearly in the introduction.

<u>Authors response:</u> Studies on short- and long-term mortality for patients with different NACA scores in HEMS are relatively rare. Especially outcome data in a HEMS population tree years after the incident. Thus, we argue that our study provides a valuable and fresh reference value for services using NACA scores. This may be useful for describing and comparing HEMS populations and as a starting point for quality improvement in these services. Moreover, this study is an example of how useful analysis can be run easily when data from both pre- and in-

hospital databases are linked in a data warehouse. The use of linked data between pre- and in-hospital services are not uncommon on a project-by-project basis. However, in our service we have established a continuous linkage between such data sources which allows us to provide continuous quality assurance initiatives. To present this, was also an aim of the study, though we absolutely acknowledge that this issue was poorly addressed throughout the whole previous submitted manuscript. In the revised manuscript we have tried to add this subject throughout and within all sections of the revised text.

- Regarding the method, there is no comparison with other regions. Moreover, there is no comparison with other scoring systems.

Authors response: *While we absolutely acknowledge the potential value of comparing our results with other regions and scoring systems, such comparisons were beyond the scope of our current study. However, we agree that future research implementing such comparisons would ensure greater external validity across systems and services. Though we firmly believe that our study containing outcome data as long as three years after an incident, may serve the purpose of a reference when comparing services and different scoring systems in the future.*

- The results are reported sometimes with absolute numbers only (table 2), which makes it hard to compare groups. In the data collection section, it is described that data on prehospital interventions and preliminary diagnosis is obtained. However, these diagnoses are not presented in any tables?

Authors response: *Thank you for your feedback on the presentation of our data. We have revised table 2 to include percentages for all categories, providing a clearer comparison between groups. Regarding the exclusion of preliminary diagnoses, we initially included them but ultimately decided to focus solely on mortality outcomes to streamline the research question. The text in the data collection section is revised to clarify this (Page 5 / lines 137-139).*

- Also, and essentially, I do not agree with the authors' conclusion in the abstract that the results validate the NACA score as a severity scoring system, since there are no ROC curves or other diagnostic plots provided.

Authors response: *We have moderated this claim and revised the conclusion in the revised manuscript.*

- Overall, I think the paper lacks an overarching purpose, the results are to a large extent expected and I find it hard to see the novelty of the study. The discussion does not lead to constructive reflection, since there are no comparisons to discuss. I recommend to rethink the aim, expand the analysis and include other regions or other scoring systems before submitting the article in a new form.

Authors response: *Thank you for your constructive feedback. We truly believe that a larger study, including multiple regions and scoring systems would be of even greater scientific value. In the setting of this study this was not possible and beyond the scope. Future research may want to expand on this and use our study for comparison. Though we have revised the text according to the constructive comments of the editor and both reviewers and we hope that these revisions have improved the aim and overall clarity of the study.*
